# Cooling the lower abdomen to reduce postpartum blood loss: A randomized controlled trial

**DOI:** 10.1371/journal.pone.0186365

**Published:** 2017-10-16

**Authors:** Yuko Masuzawa, Yaeko Kataoka, Saki Nakamura, Yukari Yaju

**Affiliations:** 1 Graduate School of Nursing Science, St. Luke’s International University, Tokyo, Japan; 2 Department of Women’s Health and Midwifery, St. Luke’s International University, Tokyo, Japan; 3 Department of Health and Social Behavior School of Public Health Graduate School of Medicine, The University of Tokyo, Tokyo, Japan; 4 Department of Nursing Statistics, St. Luke’s International University, Tokyo, Japan; TNO, NETHERLANDS

## Abstract

**Background:**

Cooling the lower abdomen is one of the Japanese traditional non-pharmacological prophylactic managements for postpartum hemorrhage. This study aimed to evaluate the effectiveness of cooling the lower abdomen in reducing postpartum blood loss compared with no intervention. In both cases, women delivered vaginally without prophylactic oxytocin in the third stage of labor.

**Methods:**

In this randomized controlled trial, the lower abdomen was cooled by placement of an 8.6°C icepack during the first 2 h after placental delivery. The primary outcome was measured as the total blood loss within 2 h after delivery. This study had 80% power at the two tails of 5% significance level to detect the mean difference (MD, 70 g) in total blood loss within 2 h after delivery between the two groups. The sample size was calculated as 144 women (72 women per group).

**Results:**

Between January and May 2016, 160 women were randomly assigned to the intervention group (cooling the lower abdomen, *n* = 81) or the control group (*n* = 79). Baseline characteristics were similar between groups, with the exception of mean blood loss during the third stage of labor. The primary outcome was not reduced by cooling, compared with no intervention (mean blood loss, 513.3 vs. 478.1 g, respectively; MD = 35.2 g; 95% confidence interval = −65.3–135.7). No adverse events occurred; however, seven (8.7%) women in the intervention group declined to continue cooling the lower abdomen because of discomfort.

**Conclusion:**

Compared with the control group, cooling the lower abdomen did not reduce the total amount of blood loss up to 2 h after delivery.

**Trial registration:**

UMIN-CTR UMIN000019834

## Introduction

Postpartum hemorrhage (PPH) is a primary cause of maternal mortality [1,2]. Although the maternal mortality rate in Japan is low, PPH accounts for about 20% of all cases of maternal death [3]. Cooling the uterus by placing an icepack on the lower abdomen is one of the standard non-pharmacological prophylactic strategies to prevent PPH in Japan; the reasoning is that cold compresses may help to contract the myometrium and decrease blood loss. Cold therapy causes blood vessels within the smooth muscles to constrict, which subsequently decreases blood flow [4]. Furthermore, blood vessels in the skin are affected by cold, resulting in somatovisceral reflex and subsequent vasoconstriction of relevant internal organs [4]. The pelvic viscera are controlled by sympathetic nerve fibers from thoracic 10 to lumbar 2 and by parasympathetic fibers from sacral 2 to sacral 4 [5]. Cold therapy on the lower abdomen and the somatovisceral reflex decreases the blood flow of the uterus; thus, cooling the uterus could prevent PPH. In a Japanese survey about management during and after the third stage of labor, cooling the lower abdomen was administered in 80% of medical facilities [6].

In a previous intervention study about cooling the lower abdomen of women in childbirth with an icepack, the surface temperature was maintained at 8.6°C for 4 h after expulsion of the placenta. The skin surface temperature of the lower abdomen was measured using a thermometer (Coretemp^®^ CM-210; TERUMO Corporation, Tokyo, Japan) thrice: just after placental delivery (before starting the intervention) and 2 and 4 h after placental delivery [7]. In the intervention group, the mean skin surface temperature was 33.2°C at placental delivery, 25.5°C at 2 h, and 22.8°C at 4 h after placental delivery. In the control group, the mean skin surface temperature was 32.5°C at placental delivery, 32.5°C at 2 h, and 32.8°C at 4 h after placental birth. In a previous observational study about the effect of cooling the lower abdomen of women in childbirth [8], the surface temperature of the lower abdomen was measured using a thermometer (Coretemp^®^ CM-210; TERUMO Corporation, Tokyo, Japan) for 105 min (at the beginning and completion of cooling). The mean skin surface temperature ranged from 22.8°C ± 3.2°C to 28.2°C ± 2.6°C. No adverse effects of cooling the lower abdomen, for example, chilblains, have been reported in previous studies. However, a previous observational study [8] reported that 8 (50%) of 16 women described feeling cold and four of those complained of discomfort caused by cooling the lower abdomen. At the planning stage of this randomized study, the mean skin surface temperature of the lower abdomen of nonpregnant women with an icepack that was covered with a towel, in accordance with the protocol of this randomized study, was measured with a thermometer (Coretemp^®^ CM-210; TERUMO Corporation, Tokyo, Japan) for 120 min. The mean skin surface temperature was 33.7°C before cooling, 26.7°C at 5 min, and 21.5°C at 10 min. Thereafter, the mean skin surface temperature was 19.5°C ± 0.8°C for 120 min.

The aim of this study was to evaluate the efficacy of cooling the lower abdomen during the first 2 h after placental delivery to reduce postpartum blood loss, compared with no intervention.

## Materials and methods

### Study design and participants

The aim of this randomized, controlled, two-armed, parallel-design trial was to verify the effect of cooling the lower abdomen during the first 2 h after placental delivery for prevention of PPH between an intervention group and an untreated control group.

This randomized controlled trial was conducted in a perinatal medical center in Tokyo, Japan, between January 2016 and May 2016. The eligibility requirements of this study were women expecting to vaginally deliver a singleton pregnancy in a hospital with cephalic presentation at or more than 34 weeks of gestation. Women with placenta previa, previous severe PPH, intrauterine fetal death, multiparity (≥4), pre-eclampsia, polyhydramnios, estimated fetal birth weight of >4,000 g, pre-pregnancy body mass index (BMI) >40, blood coagulation disorder, use of any anticoagulants, pregnancy-induced hypertension, hepatic dysfunction, placental abruption, cesarean section birth, induction and augmentation of labor with oxytocin, administration of prophylactic oxytocin during the third stage of labor, and those who did not understand Japanese were excluded from the study. Since the aim of this study was to only assess the effectiveness of cooling the lower abdomen to prevent PPH, women administered prophylactic oxytocin during the third stage of labor were also excluded.

The researchers selected eligible participants supported by midwives’ reading of the medical records. The eligible women were informed about the goal of this study both in writing and verbally during prenatal examinations at gestational week 34 or after, and all participants provided written informed consent.

The study protocol was approved by the Institutional Review Board of St. Luke’s International University (approval no. 15–062). This trial was registered with the UMIN Clinical Trials Registry (registration no. UMIN000019834).

### Randomization and masking

Participants were randomly assigned to the intervention group (cooling of the lower abdomen) or control group (no cooling intervention) at the time of placenta delivery by use of a web-based randomization system, with permuted blocks of six and stratified by parity (primipara vs. multipara). Upon placental delivery, the investigator accessed the website for group allocation. After allocation, the researcher informed the caregiver of the allocation.

Participants and caregivers could not be masked because of the nature of this intervention. The data were not masked by allocation during analysis.

### Procedures

Before recruitment to this study, the midwives and obstetricians received training, which included an explanation in writing about how to cool the lower abdomen. Early cord clamping and controlled cord traction were applied as management of the third stage of labor. The room temperature was maintained at 27°C for all participants in both groups.

The intervention method required placing on a 8.6°C icepack (ICE-NON^®^; 270 × 170 × 27 mm, 1,100 g of nonfreezing gel in a plastic bag; Hakugen Earth Co., Ltd., Tokyo, Japan) covered with a towel on the lower abdomen for 2 h using the pubic bone as a basic landmark. This cooling method is commonly implemented in clinical settings in Japan. After randomization, the midwives assessed the position and hardness of the uterine fundus through abdominal palpation and then placed the icepack on the women’s lower abdomen. Each icepack was cooled in a freezer for more than 8 h to maintain the same surface temperature during intervention. If women in the intervention group felt discomfort from the icepack on the lower abdomen, the midwives removed the icepack, and cooling was stopped.

In the no-intervention control group, no cooling of the lower abdomen was performed. Other than the intervention, women in both groups received the same management by midwives and obstetricians.

If the participants had abnormal bleeding, the midwives and obstetricians gave treatment a priority to this research, and the treatments were provided at the discretion of the individual physician in both groups.

### Outcomes

The outcomes were in line with the guidelines of the World Health Organization for the prevention and treatment of PPH [3]. The primary outcome was total blood loss up to 2 h after placental delivery (in grams), which included blood loss during the third stage of labor. The secondary outcomes were the incidence of blood loss of ≥500 and ≥1,000 g, use of therapeutic uterotonics, use of blood transfusion, postpartum anemia, transport to a tertiary emergency medical facility, any side effect of intervention, nausea, vomiting, headache, breast feeding, cooling-induced discomfort, and abdominal pain. The incidence of blood loss of ≥500 g and ≥1,000 g was assessed based on the total blood loss during the third stage of labor and the first 2 h after placenta delivery.

### Data collection

The blood loss was measured thrice, just after the placental delivery and at the first and second hour after placental delivery. Just after newborn delivery, the midwives removed the mat soaked with amniotic fluid and placed a new mat under the women’s buttocks. The new mat was kept in place until placental delivery, and the blood collected in the mat was weighed as blood loss at the third stage of labor. The midwife replaced the mat with a sanitary pad immediately after expulsion of the placenta. Blood lost during the first and second hour after placental delivery was collected with sanitary pads. The blood collected on the mat and sanitary pads was weighed on a digital scale and measured by the weight of the mat and pads after subtracting the dry weight of the same unused materials (270 and 20 g, respectively).

The pain level of each uterine contraction was measured using a vertical 100-mm visual analog scale at 1-h intervals during the first 2 h after placental delivery. The participants were instructed to mark the level of pain on a 100-mm line from none (0) to the most (100). At the same time, the level of discomfort associated with placement of the ice pack was measured using the same vertical 100-mm visual analog scale. Demographics and obstetrical characteristics were collected from medical records.

## Statistical analysis

The primary outcome of this study was the total amount of blood loss (in grams) up to 2 h after delivery. The sample size was calculated on data from a previous nonrandomization study among women who received prophylactic oxytocin in the third stage of labor [9], which showed that the mean blood loss within 2 h after delivery was decreased by 70 g (standard deviation [SD] = 150) in the intervention group, compared with the control group. This study had 80% power at the two tails of a 5% significance level to detect the mean difference (MD) of the total blood loss in the 2 h after delivery (70 g) between the two groups. Thus, the sample size was calculated as 144 women (72 women per group). No interim analysis was planned, and an independent data and safety monitoring committee were not organized because this was not a double-blind study.

The primary analysis for comparison of the two groups was by intention to treat. The secondary analysis was by per protocol. The *t*-test was used for comparison of continuous variables between the two groups, and the MD was calculated. The chi-square test was used for comparison of dichotomous variables, and the results are reported as the relative risk (RR) with the 95% confidence interval (CI). Analysis of covariance (ANCOVA) with a covariate for the amount of blood loss during the third stage of labor, which was not prespecified in the analysis, was performed to describe the effect of cooling the lower abdomen to reduce postpartum blood loss during the actual cooling process because the mean blood loss during the third stage of labor, which was a baseline characteristic, was not well balanced between the groups. Furthermore, the chi-square test was used to compare the incidence of blood loss of ≥100 g during the first 2 h after placental delivery. In Japanese clinical settings, the amount of blood loss of ≥100 g during the first 2 h after placental delivery is considered abnormal. Because of this, we set 100 g as the cutoff point. All statistical analyses were performed using SPSS software (version 24.0, IBM Corp., Armonk, NY).

## Results

**Fig 1:**
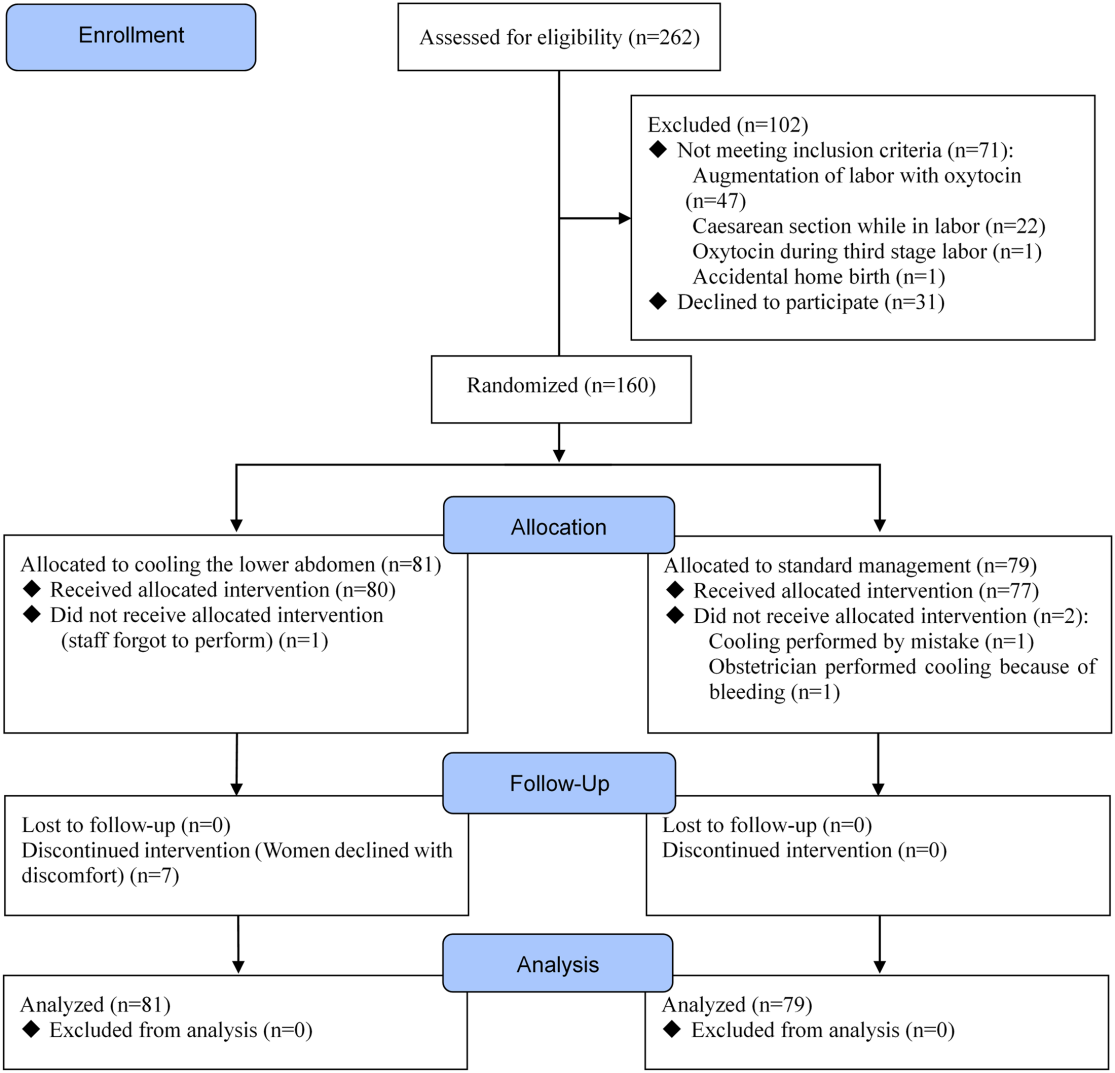
Trial flow diagram.

Of the 262 women deemed eligible, 231 consented to participate in this study and provided written informed consent between January 2016 and May 2016. When the sample size was reached, the recruitment was stopped. After the start of labor, 71 women were excluded because of the following reasons: 47 received labor augmentation, 22 gave childbirth by emergency caesarian section, one received an oxytocin injection during the third stage of labor, and one accidently delivered at home. Thus, a total of 160 women were randomly assigned to the intervention group (i.e., cooling the lower abdomen, *n* = 81) or to the control group (i.e., no intervention, *n* = 79). A flowchart demonstrating the recruitment of the study participants is shown in Fig 1. No women were lost to follow-up in the current study. The mean age of participants was 32.5 years (range: 18–46 years). Baseline characteristics were similar between groups with the exception of mean blood loss during the third stage of labor (Table 1). Furthermore, other events that occurred and might have influenced PPH were vaginal hematoma, vaginal arterial trauma, and placental abruption in the intervention group and placenta accreta in the control group. Almost all of the participants received the allocated management: 80 (98.8%) of 81 women in the intervention group received cooling of the lower abdomen and 77 (97.5%) of 79 women in the control group received no intervention. Among all participants, 139 (86.9%) were placed on a new blood collector mat before placental delivery. Although there were no adverse events, seven (8.7%) women in the intervention group declined to continue cooling the lower abdomen because of discomfort.

**Table 1:** Baseline characteristics of the study participants. Values are numbers (percentages) of women.

Characteristics	Cooling the lower abdomen (*n* = 81)	No intervention (*n* = 79)
Maternal age, mean (SD), (years)	32.5 (5.6)	32.4 (5.6)
Parity 0	36 (44.4)	33 (41.8)
1	29 (35.8)	32 (40.5)
2	14 (17.3)	11 (13.9)
3	2 (2.5)	3 (3.8)
Body mass index, mean (SD), (kg/m^2^)	21.0 (3.4)	20.6 (2.7)
Prenatal hemoglobin, mean (SD), (g/L)	11.5 (0.8)	11.4 (0.8)
Prenatal hematocrit, mean (SD), (%)	34.6 (2.3)	34.6 (2.5)
Gestational age, mean (SD), (weeks)	39.4 (1.2)	39.5 (1.2)
Length of first and second stage of labor, mean (SD), (h)	7.0 (4.6)	6.8 (4.9)
Length of third stage of labor, mean (SD), (min)	10.1 (8.3)	9.6 (7.5)
Prelabor rupture of membranes	20 (25.3) (*n* = 79)	20 (25.3)
Delivery position Supine	58 (71.6)	60 (75.9)
Lateral	17 (21.0)	13 (16.5)
Hands and knees	4 (4.9)	5 (6.3)
Squat	0	1 (1.3)
Stand	2 (2.5)	0
Instrumental delivery Vacuum extraction	2 (2.5)	3 (3.7)
Forceps delivery	0	1 (1.2)
Episiotomy	12 (14.8)	9 (11.4)
Number of women with lacerations Intact	7 (8.6)	5 (6.3)
First degree	7 (8.6)	16 (20.3)
Second degree	66 (81.5)	56 (70.9)
Third degree	1 (1.2)	2 (2.5)
Vaginal hematoma	1 (1.2)	0
Vaginal arterial trauma	1 (1.2)	0
Placenta accreta	0	1 (1.2)
Placental abruption	1 (1.2)	0
Mean blood loss at third stage (SD), g	452.7 (333.5)	396.1 (299.4)
Birth weight, mean (SD), g	3,050.1 (390.3)	3,013.8 (356.1)

The trial outcomes are shown in Table 2. All of the randomly allocated participants were included for analysis. There were no missing data. As the primary outcome, the mean total blood loss up to 2 h after delivery was not reduced in the intervention group, compared with the control group (513.3 vs. 478.1 g, respectively, MD = 35.2 g, 95% CI = −65.3–135.7).

**Table 2:** Trial outcomes by intention-to-treat analysis.

Outcomes	Cooling the lower abdomen (*n* = 81)	No intervention (*n* = 79)	Relative risk (95% CI)	Mean difference (95% CI)	*p* value
Mean total blood loss (SD), g	513.3 (333.2)	478.1 (310.1)	−	35.2 (−65.3–135.7)	0.49
Blood loss ≥500 g	36 (44.4)	28 (35.4)	1.21 (0.86–1.7)	−	0.26
Blood loss ≥1,000 g	6 (7.4)	6 (7.6)	0.98 (0.55–1.78)	−	1.00
Mean blood loss in the third stage (SD), g	452.7 (333.5)	396.1 (299.4)	−	56.7 (−42.4–155.7)	0.26
Mean blood loss during the first 2 h after placental delivery (SD), g	60.6 (51.6)	82.0 (98.6)	−	−21.5 (−46.0–3.0)	0.086
Transportation to tertiary medical facilities	0	0	−	−	−
Blood transfusion for postpartum hemorrhage	0	0	−	−	−
Therapeutic uterotonics after placental delivery	32 (39.5)	40 (50.6)	0.79 (0.58–1.09)	−	0.20
Oxytocin (5 IU)	22 (27.2)	19 (24.1)	−	−	−
Oxytocin (10 IU)	7 (8.6)	13 (16.5)	−	−	−
Oxytocin (15 IU)	2 (2.5)	6 (7.6)	−	−	−
Oxytocin (20 IU)	1 (1.2)	2 (2.5)	−	−	−
ergo-metrine	2	3	−	−	−
Mean peripartum change in hemoglobin, mean (SD), (g/L)	1.2 (1.2)	1.0 (1.0) (*n* = 78)	−	0.16 (−0.20–0.51)	0.30
Mean peripartum change in hematocrit, mean (SD), (%)	2.9 (3.8)	2.5 (3.2) (*n* = 78)	−	0.44 (−0.66–1.55)	0.42
Breastfeeding	51 (63.0)	44 (55.7)	−	−	−
Breastfeeding with formula	21 (25.9)	31 (39.2)	−	−	−
Any side effect of intervention	……	……	……	……	……
Burning pain	0	0	−	−	−
Blistering of the skin	0	0	−	−	−
Nausea	0	0	−	−	−
Vomiting	0	0	−	−	−
Diarrhea	0	0	−	−	−
Headache	0	0	−	−	−
High blood pressure	0	0	−	−	−

Intention-to-treat analysis showed that the incidence of blood loss of ≥500 g up to 2 h after delivery was not decreased in the intervention group, compared with the control group (44.4% vs. 35.4%, respectively, RR = 1.21, 95% CI = 0.86–1.7). There was no significant difference in the incidence of blood loss of ≥1,000 g between the two groups (7.4% vs. 7.6%, respectively, RR = 0.98, 95% CI = 0.55–1.78). The use of therapeutic uterotonics was higher in the control group, but there was no significant difference between groups (39.5% vs. 50.6%, respectively, RR = 0.79, 95% CI = 0.58–1.09). No participant in either group required a blood transfusion. There was no significant difference in the mean total blood loss (±SD) within 2 h between the intervention and control groups by per-protocol analysis (508.3 ± 314.8 g, *n* = 73 vs. 482.0 ± 311.4 g, *n* = 77, respectively). Also, there was no significant difference between the two groups regarding the incidence of blood loss of ≥500 g (45.2%, 33 of 73 vs. 35.1%, 27 of 77, RR = 1.18, 95% CI = 0.90–1.54), the incidence of the blood loss of ≥1,000 g (6.8%, 5 of 73 vs. 7.8%, 6 of 77, RR = 0.99, 95% CI = 0.90–1.08), or the incidence of using therapeutic uterotonics (37.0%, 27 of 73 vs. 50.6%, 39 of 77, RR = 0.78, 95% CI = 0.58–1.04).

In the intervention group, seven (8.6%) women reported discomfort with cooling the lower abdomen and declined further intervention. The subjective outcomes about pain with uterine contraction in both groups and discomfort with cooling in the control group are shown in Table 3. There were no severe outcomes, such as maternal death or transportation to a tertiary medical facility.

**Table 3:** Subjective outcomes about pain and discomfort.

Variables	Cooling the lower abdomen (*n* = 81)	No intervention (*n* = 79)
Mean pain just after placental delivery (SD)	18.7 (24.1) (*n* = 77)	19.9 (23.9) (*n* = 75)
Mean pain at 1 h (SD)	24.7 (24.5) (*n* = 77)	28.4 (23.9) (*n* = 75)
Mean pain at 2 h (SD)	21.0 (23.2) (*n* = 77)	25.3 (24.8) (*n* = 75)
Mean discomfort by putting an ice pack just after placental delivery (SD)	10.9 (21.5) (*n* = 79)	−
Mean discomfort by putting an ice pack at 1 h (SD)	13.5 (21.5) (*n* = 79)	−
Mean discomfort by putting an ice pack at 2 h (SD)	9.4 (17.8) (*n* = 74)	−

Mean total blood loss was not reduced in the intervention group, compared with the control group (513.3 vs. 478.1 g, respectively, MD = 35.2 g, 95% CI = −65.3–135.7). However, during the intervention, the mean amount of the blood loss during the first 2 h after placental delivery was lower in the intervention group than in the control group (60.6 vs. 82.0 g, respectively, MD = −21.5 g, 95% CI = −46.0–3.0). The premise was assessed using ANCOVA, which showed that cooling the lower abdomen might reduce the total blood loss (mean blood loss [± standard error], 485.8 [8.7] vs. 506.4 [8.8] g, respectively, MD = −20.6 g, 95% CI = −45.18–4.06, *p* = 0.10) and the blood loss during first 2 h after placental delivery (mean blood loss, 61.0 [8.7] vs. 81.6 [8.8] g, respectively, MD −20.56 g, 95% CI = −45.18–4.06, *p* = 0.10). Non-prespecified analysis showed that the incidence of blood loss of ≥100 g during the first 2 h after placental delivery was not decreased in the intervention group, compared with the control group (11.1% vs. 16.5%, respectively, RR = 0.94, 95% CI = 0.83–1.07).

## Discussion

In this randomized controlled trial, the principal finding was that cooling the lower abdomen did not reduce the total amount of blood loss up to 2 h after delivery. To the best of our knowledge, the current study is the first randomized controlled trial to compare cooling the lower abdomen with no intervention among women who had a vaginal delivery with no prophylactic uterotonics in the third stage of labor.

The total amount of blood loss in the intervention group was not reduced (by the *t*-test) compared with in the control group (513.3 vs. 478.1 g, respectively, *p* = 0.49), thus cooling seems to increase the amount of blood loss. However, the ANCOVA results suggested that cooling reduced total blood loss in small amounts. When limited to the intervention period, the *t*-test results showed that blood loss was reduced in the first 2 h after placental delivery by the intervention group than the control group (60.6 vs. 82.0 g, respectively). Furthermore, the ANCOVA results showed that cooling reduced blood loss in small amounts during the first 2 h after delivery.

Of these findings, the reduction in the total amount of blood loss by cooling was not found, but the results of the *t*-test showed no increase in blood loss. Although cooling may reduce the amount of the blood loss during the first 2 h after placental delivery, the effect of cooling was very small. Moreover, all participants in the intervention group reported some discomfort. Given the balance of benefits and harm, these findings indicate that cooling the lower abdomen to prevent PPH may not be useful to women who have easy access to and deliver in a medical facility.

In a Japanese nonrandomization study [9], in which subjects were given a methylergometrine injection in the third stage of labor, in the group that received cooling the lower abdomen during 2 h after neonatal delivery (*n* = 101) had a significantly lower mean total blood loss during 2 h after delivery, compared with the control group (*n* = 102; 266.3 vs. 336.5 g, respectively, *p* < 0.05). In contrast, the results of our study showed cooling the lower abdomen had little effect on reducing total blood loss. Another randomized controlled trial [10] verified that uterine cooling during cesarean section reduced total blood loss. The mean blood loss during and after the 3-h operation was significantly reduced in the uterine cooling group (*n* = 100), compared with the control group (*n* = 100; 536 vs. 756 g, respectively), likely because cooling induced uterine contractions. Cooling the uterus through the abdominal wall may not work as well as cooling the uterus directly, and the effect of applying the cold pack to the lower abdomen on the somatovisceral reflex may be small.

Considering the generalizability, the mean age of first-time mothers in Japan is 30.7 years and for a second delivery is 32.5 years, and the mean BMI among Japanese women in their 30s before pregnancy is 21.8 ± 3.7 kg/m^2^ [11]. The build and age of the study participants were similar to average Japanese women of similar ages. Hence, the findings of this study may be applicable to women having a spontaneous vaginal delivery with no use of prophylactic uterotonics during the third stage of labor.

There were some limitations to this study that should be addressed. First, in this study, it was not possible to mask group allocation because of the nature of the intervention. Therefore, there may have been some bias among clinicians. However, the primary outcome of the amount of blood loss was an objective indicator. Hence, the reliability of the primary outcome was not threatened by nonmasking. Second, even though this was a randomized controlled trial and the amount of blood loss during the third stage of labor was one of the baseline characteristics and affected the total amount of blood loss, the two groups were not equal at baseline. Furthermore, the most blood loss occurred in the third stage of labor, which was prior to intervention. Therefore, to increase precision, it is necessary to provide the inclusion criteria parameters of blood loss during the third stage of labor. Finally, the primary outcome of this study was the amount of total blood loss. Further research is needed in which the primary outcome is the incidence of blood loss of >1,000 ml, which is one of the important outcomes, and with a larger sample size.

## Conclusion

Cooling the lower abdomen did not decrease blood loss among the women who had vaginal delivery with no prophylactic uterotonics in the third stage of labor. There was no decrease in the number of women who had PPH and severe PPH in the cooling group compared with the control group. Cooling the lower abdomen is one of the unique non-pharmacological prophylactic managements for PPH in Japan, but women felt discomfort with cooling and insufficient effectiveness in reducing the amount of blood loss was observed. Cooling the lower abdomen may not be routinely useful for reducing blood loss in the first two hours after delivery for women who deliver in the medical facilities.

## Supporting information

S1 Table CONSORT 2010 checklist. (DOC)

S1 File Data set. (XLSX)

S1 Text Original study protocol. (PDF)

S2 Text Study protocol in English. (PDF)
