# Supplementary material for: Cooling the lower abdomen to reduce postpartum blood loss: A randomized controlled trial
Source: PLoS One. 2017 Oct 16;12(10):e0186365. doi: 10.1371/journal.pone.0186365 (PMC5642889; doi:10.1371/journal.pone.0186365)
Supplement: S1 Text — (PDF) [file pone.0186365.s003.pdf]

2015 年 11 月 6 日

研究計画書

冷却材を用いて下腹部を冷やすことによる分娩後出血の予防効果の検証：
ランダム化比較試験
Cooling the Lower Abdomen for Preventing Postpartum Hemorrhage：
A Randomized Controlled Trial

増澤祐子
片岡弥恵子

序論

I.　研究の背景

分娩後出血は妊産婦死亡の主要な原因の 1 つである (AbouZahr, 2003; Say et al., 2014)。妊産婦死亡率の低いわが国においても，2013 年の妊産婦死亡の原因の約 20%を分娩後出血は占めている (母子衛生研究会, 2015)。分娩後出血の予防は世界的に取り組むべき重大な問題であり，有効性の高いケアの確立が急務である。

しかし，分娩後出血の予防介入方法の中には未だ有効性が検証されていないものも多い。そのひとつである下腹部を冷やすという方法は，日本で古くから助産師が実践している分娩後出血に対する介入方法である。冷罨法により血管が収縮し血流が減少することにより止血効果がもたらされ，冷却部位だけでなく，体性内臓反射によって冷却部位と同じ神経支配となる臓器の血流も減少するとされている (Harmer, 1957)。

分娩第 3 期以降に実施しているケアの調査 (Kataoka, Shimizu, Yaju, Eto, & Horiuchi, 2015) によると，冷却材を下腹部に置いて冷やすことは，病院や診療所では約 8 割の施設で実施されている。冷却材を下腹部に置いて冷やすことの分娩後出血に対する効果について検討を試みた研究はこれまで実施されている (藤田, 真鍋, & 諸岡, 1994; 林, 稲垣, 森田, 真野, & 天野, 1995; 本道, 石塚, & 菊池, 1993; 松岡, & 三宮, 2002; 大隅, & 堀内, 2007; 大塚ら, 1990;佐藤, 野呂, & 中山, 1984)。しかし，これらは観察研究や非ランダム化比較試験などの研究デザインであるため，交絡因子やバイアスを検討する必要があり，さらに子宮収縮薬の投与など冷罨法以外の分娩後出血に対する予防介入も行われている。これまでに，実際に多くの臨床現場で下腹部を冷やす際に使用されている不凍ゲルで生成されたアイスノン® (白元，270×170×27mm, 1,100g) を置いて下腹部を冷やすことの分娩後出血の予防効果の有効性について，ランダム化比較試験での検証は行われていない。

II.　研究の目的

本研究は，ランダム化比較試験にて，薬剤を用いない分娩後出血の予防介入として行われている，アイスノン®を置いて下腹部を冷やすことの有効性を検証することを目的とする。

方法

I. 研究デザイン

本研究はアイスノン®を置いて下腹部を冷やすことによる分娩後出血の予防効果の検証を目的とし，アイスノン®を置いて下腹部を冷やす介入群と，何もしない対照群の 2 群を設定するランダム化比較試験である。

II.　研究参加者の対象

研究協力施設は，関東圏にあるルチーンで分娩第 3 期に子宮収縮薬を使用していない医

療施設 (総合病院，診療所) 1，2 ヶ所程度とする。

1. **組み入れ基準**

妊娠経過が順調な妊娠 34～42 週未満の単胎/頭位の児の経膣分娩を予定する女性。

2. **除外基準**

除外基準は，以下とする。

同意が得られなかった場合，前置胎盤，分娩後出血既往 (1,000ml 以上の出血)，子宮内胎児死亡，4 経産以上，子癇，羊水過多，胎児推定体重 4,000g 以上，非妊娠時 BMI 40 以上，血液凝固障害，抗凝固薬の内服，妊娠高血圧症候群，肝機能障害，常位胎盤早期剥離，緊急帝王切開，分娩第 3 期の予防的子宮収縮薬の投与を受けた場合，日本語の読み書きができない場合。

III. 割付方法と盲検化

割り付けは，研究参加者の胎盤娩出時に実施する。ウェブサイトで管理される，産歴を要因とする層別ブロック法を用いた中央割付を行う。ブロックサイズは 6 とする。介入の性質上，研究参加者，介入者 (研究協力施設の助産師) は盲検化することができない。

研究参加者の胎盤娩出時にウェブサイトへアクセスし，介入群と対照群のどちらの群に割り付けとなるかを確認する。割り付けが決定したら，研究参加者の分娩に携わっている研究協力施設の助産師へ伝える。

IV. 介入方法

本研究実施前に，研究協力施設の産科医師と分娩室の助産師とミーティングを行い，アイスノン®を置いて下腹部を冷やす方法について実際に示す。

1. **介入群への介入方法**

介入群に割り付けとなった場合，介入者である研究協力施設の助産師が，まず，子宮底の位置を腹部からの触診で確認する。子宮底の位置を確認した後，子宮体部にアイスノン®があたるよう，恥骨を起点として，恥骨と子宮底の間の下腹部にカバーで包んだアイスノン®を研究協力施設の助産師がのせる。胎盤娩出後 2 時間の間，このアイスノン®を置いて下腹部を冷やすことを実施する。

下腹部を冷やすことに使用するアイスノン®は，あらかじめ冷凍庫にて 8 時間以上冷却しておく。アイスノン®を置いて下腹部を冷やすことが不快で，研究参加者に中止の希望がある場合には，アイスノン®を置いて下腹部を冷やすことを中止する。

2. **対照群への対応方法**

対照群に割り付けとなった場合，アイスノン®も何も下腹部にのせない。

3. **介入方法の検討**

本研究における介入方法は，臨床現場において実践されているものであり，既存研究にて冷罨法実施中の下腹部皮膚表面温度についての結果が示されているため，パイロット研究は実施していない。

室温 27 度の環境で，タオルで包み，表面温度が 8 度となった冷凍したアイスノン®を用いて，胎盤娩出後 4 時間，下腹部冷罨法を実施した介入研究 (林ら, 1995) では，介入前 (分娩直後) と 2 時間後，4 時間後の 3 時点で皮膚表面温度をサーモグラフィーにて測定している。冷罨法実施群は，介入前は平均 33.2 度，2 時間後は平均 25.5 度, 4 時間後は 22.8 度だった。冷罨法を実施していない群は，介入前は平均 32.5 度，2 時間後は 32.5 度，4 時間後は 32.9 度だった。また，別の観察研究 (大隅ら, 2007) では，冷罨法開始前から開始後 105 分までの期間継続的に皮膚表面温度を体温計 (コアテンプ®CM-210, テルモ株式会社, 東京) を使用して計測を行っており，22.87±3.22 度～28.18±2.59 度で推移していた。先行研究において凍傷などの身体的影響についての報告はない。冷罨法による下腹部の冷感と不快感を聴取した観察研究 (大隅ら, 2007) によると，冷感を感じていたのは半数 (8/16 名) で，そのうちの半数 (4/8 名) が不快と感じていた。

本研究にて使用するカバーで包んだアイスノン® (表面温度 8.5 度) を使用し，非妊娠女性の下腹部に冷罨法を実施した際の下腹部皮膚表面温度を体温計 (コアテンプ®CM-210，テルモ株式会社，東京) を用いて測定した。冷罨法開始前の下腹部皮膚表面温度は 33.7 度で，下腹部の冷罨法開始 5 分後には 26.7 度，10 分後には 21.5 度まで低下した。その後，冷罨法開始 120 分後までは平均 19.5±0.8 度で推移していた。

4. 両群における分娩期第 3 期のケア

両群ともに, 分娩室の室温は母児にとって快適な温度である 27 度に空調を設定する。また，胎盤娩出方法，臍帯結紮の方法は，研究協力施設において，ルチーンで行っている方法に従う。

5. 出血時の対応

異常出血が生じた場合には，研究協力施設の行っているプロトコルに従い，産科医師や助産師の判断を基に実施される医療を研究よりも優先する。

V. アウトカム

WHO は，分娩後出血に対する予防と治療に関するガイドライン (WHO, 2012) で，意思決定に重大なアウトカムを定めている。本研究のアウトカムはこのガイドラインを参考に設定した。

1. プライマリアウトカム

・分娩後 2 時間の総出血量 (グラム)

2. セカンダリアウトカム

・分娩後出血 (分娩後 2 時間までの総出血量 500g 以上) の有無
・分娩後 2 時間の治療を目的とした子宮収縮薬の使用の有無
・分娩後出血 (分娩後 2 時間までの総出血量 1000g 以上) の有無
・産前，産後の貧血に関する血液データ (ヘモグロビン，ヘマトクリットなど) の値とその変化
・輸血の有無
・高次医療機関への搬送の有無
・分娩後 2 時間以降より入院中の出血量と分娩後出血に対する治療の有無と種類
・冷罨法による凍傷などの身体的影響の有無
・冷罨法以外の分娩後出血に対する治療による副作用の有無 (頭痛，吐き気，下痢，腹痛，血圧上昇など)
・分娩後 2 時間の後陣痛と冷罨法による不快感の有無と程度
・入院中の授乳の状況

VI. データ収集

1. データ収集期間

倫理審査承認後～2016 年 8 月頃を予定している。

2. データ収集内容と方法

研究協力施設の施設代表者と看護管理者に対し，説明書を用いて文書と口頭で研究協力の依頼を行う。研究協力施設のスタッフが医療記録を閲覧し，その補助のもと，研究者が研究参加者の対象の組み入れ基準に合う女性を選定する。研究参加者の対象の組み入れ基準に合う女性に対し，妊娠 34 週以降の妊婦健診時に，研究者が本研究の内容について文書と口頭で説明し，協力の依頼を行い，同意が得られた場合には書面に承諾を得る。承諾を得られた場合でも，陣痛発来後，緊急帝王切開となった場合，児娩出後に子宮収縮薬の予防的投与を受けた場合は本研究の対象から除外されることも説明する。また，研究結果公表前であれば，書面にて研究参加同意の撤回が可能なことについて伝える。

1) デモグラフィックデータ

年齢，身長，体重，妊娠分娩歴，過去の分娩時の出血量を，研究協力施設のスタッフの医療記録閲覧補助のもと，医療記録より収集する。

2) 妊娠･分娩に関する医療データ

分娩週数，児の出生体重，分娩経過，分娩様式，会陰裂傷の有無･程度と処置，分娩後 2 時間のバイタルサインズを，研究協力施設のスタッフの医療記録閲覧補助の

もと，医療記録より収集する。

3) **分娩後 2 時間の子宮収縮に伴う痛みの程度**

女性が自覚した痛みの程度を「0：まったく痛くない」から「100：きわめて痛い」の Visual Analogue Scale を用いて測定する。質問紙に記載された 100mm の線上に自覚した痛みの程度を，胎盤娩出直後，分娩後 1 時間， 2 時間の時点の合計 3 回記載してもらう。

4) **分娩後 2 時間の下腹部を冷やすことによる不快感の程度**

介入群に割り付けられた女性が自覚したアイスノン®を置いて下腹部を冷やすことによる不快感の程度を「0：まったく不快でない」から「100：きわめて不快」の Visual Analogue Scale を用いて測定する。質問紙に記載された 100mm の線上に自覚した不快感の程度を，胎盤娩出直後，分娩後 1 時間， 2 時間の時点の合計 3 回記載してもらう。

5) **分娩後 2 時間までの出血量**

児娩出後に速やかに女性の臀部の下にシートを敷き，羊水の混入を避けた分娩直後の出血量を計測する。胎盤娩出後にパットをあて，分娩後 1 時間， 2 時間の時点でパットを交換し，そのパットより出血量をデジタルスケールにて，グラムで計測する。研究協力施設の助産師が合計 3 回出血量の計測を実施し，分娩後 2 時間までの総出血量を分娩後出血量とする。

6) **その他の項目**

分娩後 2 時間の子宮収縮の程度 (硬度と大きさ)，分娩後出血に対する治療の有無，その種類と副作用，下腹部を冷やすことによる副作用の有無，産前，産後の血液検査データの値と貧血に対する治療の有無と治療内容，分娩後 2 時間以降より入院中の子宮収縮の程度，出血量と分娩後出血に対する治療の有無と種類，入院中の授乳状況について，医療記録より収集する。

プロトコルの遵守については，研究者もしくは研究補助者が介入時に観察する。児娩出直後にシートを挿入したか，アイスノン®を置いて下腹部を冷やすことを実施した時間，プロトコルを遵守した下腹部を冷やす方法であったかを，データシートに記載する。

VII. 対象者数

本研究のプライマリアウトカムを分娩後 2 時間の総出血量とする。先行研究 (佐藤ら，1984) を参考に，$\alpha=0.05$，$\beta=0.2$ で計算した。アイスノン®を置いて下腹部を冷やす介入群と何もしない対照群の分娩後 2 時間の総出血量の平均値の差を 70g，SD=150g とし，サンプルサイズを算定した。1 群は，$2(1.96+0.84)^2\times150^2/70^2=15.68\times4.5=71.9=72$ 人の為，両群合わせて 144 人をサンプルサイズとする。

緊急帝王切開になる女性の割合が18%，分娩第3期に子宮収縮薬投与を受ける女性が40%と考え，360人程度をリクルート予定。

VIII. データ分析方法

データシート，質問紙の各項目について基本統計量 (中央値と範囲，平均値と標準偏差，度数，割合) を算出する。プライマリアウトカムとセカンダリアウトカムについては，Intention-to-treat analysis で2群の比較を行う。副次的分析として，per-protocol analysis も行う。アウトカムが連続変数の場合は平均差 (Mean difference) を2区分変数の場合は，リスク差 (risk difference) と対照群をリファレンスとした時の相対危険度 (relative risk) を計算し，どちらの変数においても95%信頼区間を算出する。データ分析には，統計ソフトSPSS version 22.0 for Windows を使用し，有意水準は5%とする (両側検定)。

1. 主要な統計解析

   介入群と対照群の分娩後2時間の総出血量の差についてt検定を行う。

2. 副次的な統計解析

   介入群と対照群の分娩後出血，子宮収縮薬使用の有無についてχ二乗検定を行う。

IX. 倫理的配慮

本研究計画書は，文部科学省・厚生労働省「人を対象とする医学系研究に関する倫理指針」に沿って作成する。

研究協力の依頼にあたり，以下の内容を文書および口頭で説明し同意を得た上で行う。

1. 本研究の参加，参加を継続するかどうかは，研究参加者の自由意思によるものであること。研究結果公表前であれば，研究協力の同意撤回ができること。
2. 研究参加者の意思により，参加をしない場合，途中で研究協力の同意を撤回した場合でも，いかなる不利益を被ることはないこと。研究協力の同意を撤回した場合には，その時点までに収集したデータは研究データとして使用するが，データ破棄の希望があれば，データは破棄できること。
3. 研究に参加していても，通常通り，研究協力施設の医師・助産師から分娩後2時間の母児のケアは受けられること。また，児の授乳を妨げることはないこと。
4. 本研究に協力することによるメリットは，研究参加者自身が直接得る利益は少ないかもしれないが，本研究の結果は女性の分娩後出血の予防ケアの確立に活用されること。本研究に協力することによるデメリットは，質問紙の回答に労力を要すること。また，介入群の場合，分娩後2時間の間，アイスノン®を置いて下腹部を冷やすことによる不快感を生じる可能性があること。

5. 下腹部を冷やすことを中止したい場合は，中止できる。通常通り，研究協力施設の助産師は下腹部を冷やしている際に凍傷などの身体的影響が生じていないかの観察を行う。これまで，分娩後の下腹部を冷やすことにより身体的影響が生じた事例は報告されていない。万が一，凍傷などの身体的影響が生じた場合には，適切な治療が受けられるよう研究協力施設の医師と連携をとること。本研究の介入によるアイスノン®を置いて下腹部を冷やすことで身体的影響が生じた場合には，提供される治療等は研究者が加入している保険にて補償されること。その他金銭での補償は行わないこと。
6. データシートは個人名を記載せず，符号化を行う。研究参加者の匿名性を常に確保し，個人情報・プライバシーの保護を行うこと。
7. データシートは，施錠された場所に保管を行い，研究者のみが利用できるよう厳重に管理を行う。収集されたデータは，匿名性を守り，パスワードで保護されたパソコンおよびフラッシュメモリーに保存する。なお，研究者のみがアクセスできるようパスワードの管理は徹底する。
8. 個人情報を匿名化した情報は，本研究の目的以外では使用しないこと。また，本研究の終了を報告した日から 5 年間，もしくは学術誌や学会に公表した後 3 年間のいずれか長い期間は保管するが，その他の情報は研究終了後に破棄されること。
9. 本研究は，研究計画書の段階で聖路加国際大学研究倫理審査委員会において承認を得られた後に実施する。（承認番号：15-062）
10. 聖路加国際大学研究倫理審査委員会において本研究の承認を得た後，研究協力施設に研究協力の依頼を行う。研究協力の同意を得た後，研究協力施設の倫理審査委員会にて承認を得られた後に本研究を実施する。
11. 聖路加国際大学研究倫理審査委員会において本研究の承認を得た後，研究に先立って UMIN 臨床試験登録システム (UMIN-CTR) に本研究の概要について登録を行う。(試験 ID:UMIN000019834/登録日:2015/12/01)
12. 聖路加国際大学研究倫理審査委員会において本研究の承認を得た後，日本看護学校協議会共催会総合補償制度である Will と e-kango に加入する。
13. 本研究は，平成 26 年度文部科学省科学研究費助成事業 挑戦的萌芽「日本で継承されてきた助産ケアの探索と標準化に向けた再構築」（課題番号：26670993）の一部助成を受け実施する。

2015 年 11 月 13 日

聖路加国際大学研究倫理審査委員会結果通知

准教授　片岡　弥恵子　先生

聖路加国際大学
学長　井部　俊子

あなたの指導する下記から提出された研究計画書について、2015 年 10 月 20 日、2015 年度第 6 回研究倫理審査委員会において検討の結果、条件付き承認となった計画書の修正事項が確認されました。結果を以下の通りお知らせします。

記

承認番号：１５－０６２

増澤祐子（博士後期課程 2 年　助産学　指導教員：片岡弥恵子准教授）

研究課題：下腹部冷罨法による分娩後出血の予防効果の検証：ランダム化比較試験

審査結果：承認

以上
