# Supplementary material for: Cooling the lower abdomen to reduce postpartum blood loss: A randomized controlled trial
Source: PLoS One. 2017 Oct 16;12(10):e0186365. doi: 10.1371/journal.pone.0186365 (PMC5642889; doi:10.1371/journal.pone.0186365)
Supplement: S2 Text — (PDF) [file pone.0186365.s004.pdf]

November 6, 2015

Study Protocol

Yuko Masuzawa
Yaeko Kataoka

Introduction

Background

Postpartum hemorrhage (PPH) is one of the leading cause of maternal mortality (AbouZahr, 2003; Kassebaum et al., 2014). In spite of Japan’s low maternal mortality rate, PPH accounts for about 20 % of all cause of maternal death (Mothers' and Children's Health Organization, 2015). As a significant medical threat, effective strategies for the prevention and treatment of PPH are essential to decrease maternal mortality rates worldwide.

However, some of prophylactic interventions for PPH have not been evaluated. Cooling the uterus, through putting an icepack on the women’s lower abdomen, is one of the unique non-pharmacological prophylactic strategies for PPH in Japan; the reasoning is that cold compresses may help contract the myometrium and decrease blood loss. Cold therapy causes blood vessels within the smooth muscles to constrict and decrease the blood flow (Harmer, 1957). Furthermore, blood vessels in the skin are affected by cold, resulting in somato-visceral reflex and subsequent vasoconstriction of relevant internal organs (Harmer, 1957).

In a Japanese survey about management during and after the third stage of labor, cooling the lower abdomen was provided in 80% of medical facilities (Kataoka, Nakayama, Yaju, Eto, & Horiuchi, 2015). There was some evidence about cooling the lower abdomen for postpartum blood loss, but was limited to observational studies (Fujita, Manabe, & Morooka, 1994; Matsuoka & Sannomiya, 2002; Osumi & Horiuchi, 2007) or non-randomization studies (Hayashi, Inagaki, Morita, Mano, & Amano, 1995; Hondo, Ishizuka, & Kikuchi, 1993; Otsuka et al., 1990; Sato, Noro, & Nakayama, 1984), and the findings were the opposite. Many facilities provided cooling the lower abdomen for preventing PPH, but the effect of cooling the lower abdomen for prevention of PPH has not been evaluated.

Purpose of This Study

The aim of this study is to evaluate cooling the lower abdomen during the first two hours after the placental delivery to reduce postpartum blood loss compared with no intervention.

Methods

Study Design

This randomized controlled trial compare the intervention group who has cooling the lower abdomen during the first two hours after the placental delivery with the comparison group not cooling the lower abdomen, in order to verify the effect of

prevention for PPH.

Participants

This randomized controlled trial is conducted in a perinatal medical center in Tokyo, Japan. The eligibility requirements of this study are women expecting to deliver vaginally at the hospital, and to have a singleton pregnancy with cephalic presentation at or more than 34-week gestation. This study exclude women with placenta previa, previous severe PPH, intrauterine fetal death, multiparity (four or more), pre-eclampsia, polyhydramnios, estimated fetal birth weight over 4,000 g, pre-pregnancy BMI over 40, blood coagulation disorder, taking any anticoagulants, pregnancy-induced hypertension, hepatic dysfunction, placental abruption, caesarean section birth, induction and augmentation of labor with oxytocin, administration of prophylactic oxytocin during the third stage of labor and those who do not understand Japanese.

The participants will be recruited between December 2015 and August 2016.

Randomization and Masking

Participants are randomly assigned to a cooling the lower abdomen group or no intervention group at the time of placenta delivery, by use of a web-based computer site, with permuted blocks of six and stratified by parity. When the participants deliver the placenta, the investigator access the website and check the allocation. After the decision of the allocation group, the researcher tell the caregiver about the allocation.

Procedures

Before starting recruitment for this study, the midwives and obstetricians are trained and received an explanation with a document about how to cool the lower abdomen.

The intervention method is putting 47.3 °F icepack (ICE-NON®; Hakugen Earth CO., LTD., Tokyo, Japan, 270×170×27mm, 1,100 g of nonfreezing gel in a plastic bag) covered with a towel on the lower abdomen for two hours, using the pubic bone as the basic point. After the randomization, midwives assess the position and hardness of the fundus of the uterus through abdominal palpation, and put the icepack on the women's lower abdomen. This icepack is cooled in a freezer for more than eight hours, in order to keep the same surface temperature of the icepack during intervention. If women in the intervention group feel discomfort about putting an icepack on her lower abdomen, midwives remove the icepack and cooling is stopped.

In the previous intervention study about cooling the lower abdomen of women in childbirth with the icepack, surface temperature was 47.3 °F, for four hours after expulsion of the placenta (Hayashi et al., 1995), the skin surface temperature of the participants' lower abdomen was measured by a thermometer (Coretemp® CM-210;

TERUMO Corporation, Tokyo, Japan) three times: just after placental delivery (before starting the intervention), two hours and four hours after the placental delivery. In the cooling group, the mean skin surface temperatures were 91.76 °F (at placental delivery), 77.9 °F at two hours, and 73.0 °F at four hours after placental birth. In the non-cooling group, the mean skin surface temperatures were 90.5 °F (at placental delivery), 90.5 °F at two hours, and 91.2 °F at four hours after placental birth. In the other observational study about cooling the lower abdomen of women in childbirth (Osumi & Horiuchi, 2007), the researcher measured the surface temperature of the participants' lower abdomen using by a thermometer (Coretemp® CM-210; TERUMO Corporation, Tokyo, Japan) for 105 minutes (at the beginning and completion of cooling). The mean skin surface temperatures were 73.0 ± 37.7 to 82.7 ± 36.6 °F. Any adverse effect of the cooling the lower abdomen, for example chilblains, had never been reported in previous studies. However, an observational study (Osumi & Horiuchi, 2007), the researchers found that women described feeling cold and discomfort caused by cooling the lower abdomen; eight people of 16 participants felt the cold and of those, four felt discomfort.

At the planning of this randomized study, the mean skin surface temperatures of lower abdomen of non-pregnant with the icepack, which cooled and covered with a towel in accordance to the protocol of this randomized study, were measured by a thermometer (Coretemp® CM-210; TERUMO Corporation, Tokyo, Japan) for 120 minutes. The skin surface temperatures were 92.6 °F (before cooling), 80.0 °F at five minutes, and 70.7 °F at 10 minutes. After that, the mean skin surface temperatures were 67.1 ± 33.4 °F for 120 minutes.

In the control group, women have no intervention, which is not putting the ice pack on the lower abdomen and no cooling.

If the participants have abnormal bleeding, midwives and obstetricians give treatments priority to this research and the treatments are provided at the discretion of the individual physician in the both groups.

Outcomes

The outcomes are decided in line with the critical and important outcomes for decision making from the guidelines of the WHO about prevention and treatment of PPH (WHO, 2012).

The primary outcome is the amount of total blood loss (grams) within the two hours after the newborn delivery. The secondary outcomes are the incidence of blood loss of 500 g or more and 1,000 g or more, use of therapeutic uterotonics, use of blood transfusion, postpartum anemia, transport to tertiary emergency medical facility, any side effect of intervention, nausea, vomiting, headache, breast feeding, discomfort at

cooling, and abdominal pain.

Data Collection

Permission for this study is sought in writing from the representative and director for nursing of the perinatal medical center or orally. The researcher select eligible participants support by midwives' by reviewing the medical records. The eligible women are informed about this study by both written information and verbally during their prenatal examination of at least 34 weeks' gestation. The women agree to participate by signing a consent form. Women are informed that, in case of emergency caesarean section, induction and augmentation of labor with oxytocin, and administration of uterotonics during the third stage of labor, they would be excluded from this study even though they agreed to participate. In addition, they are told they can retract their consent without penalty by using the withdrawal document.

Demographic data. Demographic data (age, height, weight, parity, and history of PPH), and obstetrical characteristics (gestational age, neonatal birth weight, mode of the delivery, presence of perineal laceration, vital signs during the first two hours after the delivery), were collected.

Pain levels with uterine contraction. Pain levels with uterine contraction are measured by using a vertical 100-mm visual analogue scale at one-hour intervals during the first two hours after placental delivery. Participants mark their pain level on the 100-mm line indicating pain from none (0) to the most (100).

Ice-pack discomfort levels. Feelings of discomfort from the ice pack are measured by using a vertical 100-mm visual analogue scale at one-hour intervals during the first two hours after placental delivery. Participants mark their discomfort level on the 100-mm line indicating discomfort from none (0) to the most (100).

Blood loss within two hours after delivery. Just after newborn delivery, the midwives remove the mat soaked with amniotic fluid and place a new mat under the women's buttocks. This new mat is placed until placental delivery, and blood in the mat is weighted as blood loss at the third stage of labor. The midwife replace the mat with a sanitary pad immediately after the expulsion of the placenta. Lost blood is collected with sanitary pads the first and second hour after placental delivery. The blood loss with mat and sanitary pads are weighted on a digital scale, and measure by the weight of mat and pads after subtracting the dry mat weight of 270 grams and the dry pad weight of 20 grams.

Other variables. Other variables are collected from participants' medical record; strength of uterine contractions, treatment for PPH and adverse effect from the treatment, adverse effect of the cooling, blood test data about anemia before and after the

delivery, absence of treatment for anemia, and breastfeeding during hospitalization.

The researcher monitor the adherence to protocol about putting the mat, the duration of the ice pack, and the method of cooling.

Statistical Analysis

The primary outcome is the amount of total blood loss (grams) within the two hours after the newborn delivery. The sample size is calculated on data from a previous study (Sato et al., 1984). This study has 80% power at the 5% significance level to detect the mean difference of the total blood loss of 70 grams between the two groups. Thus, the sample size is calculated as 144 women (72 women per group).

The primary analysis is by intention to treat for comparing two groups. The secondary analysis is by per-protocol. For comparison between the two groups, the t-test is used for continuous variables and mean difference is calculated. The chi-square test for dichotomous variables is applied and relative risk with 95% CI is calculated.

The statistical analysis is performed using SPSS software (version 24.0).

Ethical Considerations

The "Ethical Guidelines for Medical and Health Research Involving Human Subjects" guided the study protocol:

- Whether to participate or participate in this research is to be based on the will of the research participants. Before publication, it is possible to withdraw the consent of research participation.
- If women do not participate, or withdrew their consent of research participation during the study, they do not suffer any disadvantage. If women withdrew their consent, the research data collect up to that point will be discarded if the women desire to discard data.
- Even if women participate in the research, as usual, women can receive maternal care for two hours after parturition from obstetricians and midwives at the research cooperation facility. Participation in the research do not interfere with breast-feeding.
- Those participating in the research may not experience any benefits by participating however the results of this study will be utilized to establish preventive management for postpartum hemorrhage. The disadvantage of participating with this research is, that it requires participant's effort to answer the questionnaire. Also, in the case of the intervention group, there is a possibility of discomfort due to cooling the lower abdomen during the two hours after the delivery.
- If women want to stop cooling the lower abdomen, they can stop. As usual, the midwives of the research cooperation facility observed whether physical influences

such as frostbite had occurred while cooling the lower abdomen. There had been no reported cases of physical influences caused by cooling the lower abdomen after parturition. In the unlikely event of a physical influence such as frostbite, there will be consultation with a physician at the research cooperation facility so that appropriate treatment could be received. In the event that, a physical condition occurs due to the intervention of this research, the treatment provide will be compensated by the insurance covered by the researcher, but not compensate by money to the participant.

- The data sheets do not identify the participant's name and performs encoding. Anonymity of research participants and protect personal information and privacy is always ensured.
- The data sheets are stored in the locked place and strictly managed so that only the researcher can use it. The collected data is protected so that anonymity is maintained and stored in a password protected personal computer and flash memory. In addition, password management is thorough so that only the researcher could access it.
- Information that anonymized personal information is not used for purposes other than this research. Also, it shall be kept for five years from the date of reporting the end of this research, or three years after publication to academic journals or academic societies, whichever is longer, but other information is destroyed after the study is over.
- The researcher's Institutional Review Board approved the research (No. 15-062).
- The research cooperation facility's Institutional Review Board approved the research.
- This trial is registered with UMIN-CTR (ID: UMIN000019834).
- This study is insured by; Will and e-kango which is the comprehensive compensation system of the Japan Nursing School Council Co-organizing Society.
- JSPS KAKENHI Grant Number 26670993 supported this study.
